# Impact of Sodium-Glucose Cotransporter-2 Inhibitors on Cardiovascular Function and Residual Kidney Function in Haemodialysis Patients: A Three-Month Follow-Up

**DOI:** 10.7759/cureus.95876

**Published:** 2025-11-01

**Authors:** Mahmoud Abdelaziz, Mohammed Abdel Hassib, Tamer Fouad, Ibrahim F. Said, Attia M. Shokr, Abdelrahman E Metwally, Ahmed S Abdelaziz, Ahmed M Elbeny, Abdelaal A. Elkhouly, Mostafa E Soltan, Ashraf A Alamir, Mohammad Eltahlawi

**Affiliations:** 1 Cardiology, Zagazig University, Zagazig, EGY; 2 Internal Medicine, Faculty of Medicine, Al-Azhar University, Cairo, EGY; 3 Cardiology, Faculty of Medicine, Al-Azhar University, Cairo, EGY; 4 Cardiology, Islamic Cardiac center, Al-Azhar University, Cairo, EGY

**Keywords:** cardiovascular risk, diastolic dysfunction, haemodialysis, residual renal function, sglt2 inhibitors

## Abstract

Chronic kidney disease (CKD) patients are at high risk for cardiovascular morbidity and mortality. Sodium-glucose cotransporter-2 inhibitors (SGLT2i) have shown promising benefits in the management of cardiovascular and renal outcomes. While SGLT2i are not approved for use in patients undergoing haemodialysis (HD), emerging evidence suggests that SGLT2i may offer cardioprotective and nephroprotective effects in patients on kidney replacement therapy. This study aims to evaluate the effects of SGLT2i on both cardiovascular status using transthoracic echocardiography and residual kidney function in HD patients over a three-month period. This prospective single-arm intervention study enrolled 40 maintenance HD patients from a tertiary care centre. Participants received empagliflozin 10 mg once daily for 12 weeks alongside standard HD therapy. Comprehensive assessments were performed at baseline and study termination, including (1) transthoracic echocardiography with measurements of left ventricular filling pressures (E/e′ ratio and left ventricular end-diastolic pressure (LVEDP)), chamber dimensions, and systolic/diastolic function according to the American Society of Echocardiography guidelines; (2) biochemical analyses of renal function-estimated glomerular filtration rate (eGFR), inflammation-high-sensitivity C-reactive protein (hs-CRP), and metabolic parameters; and (3) clinical evaluation of functional status (New York Heart Association classification) and haemodynamic parameters. Following 12 weeks of SGLT2i therapy, participants demonstrated significant improvements across multiple cardiorenal parameters. Echocardiographic changes included reduced left ventricular filling pressures (E/e′: -17.1%; LVEDP: -3.2 mmHg; both: p < 0.001) and favourable cardiac remodelling (left atrial volume index: -11.8%; left ventricular end-diastolic diameter: -4.4%; both: p < 0.001) with preserved systolic function. Systemic benefits encompassed blood pressure reduction (-10/-4 mmHg, p < 0.01), decreased inflammation (hs-CRP: -38%; p = 0.005), and improved albuminuria (albumin-to-creatinine ratio: -80 mg/g; p = 0.022). Functional capacity improved in 30% of patients (p = 0.008), with modest renal benefit (eGFR: +0.8 mL/min/1.73m²; p = 0.087). We conclude that SGLT2i therapy in HD patients was associated with improved diastolic function, reduced inflammation, and promoted reverse cardiac remodelling without compromising systolic function, while demonstrating an acceptable safety profile. These findings support further randomised trials to confirm cardiovascular and residual renal benefits in end-stage kidney disease.

## Introduction

Cardiovascular disease (CVD) remains the leading cause of mortality among patients with chronic kidney disease (CKD), accounting for approximately 40-50% of all deaths in this population, particularly those undergoing haemodialysis (HD) [1]. Despite therapeutic advances, this population faces persistently high cardiovascular risk due to progressive renal dysfunction and limited evidence-based interventions. Sodium-glucose cotransporter-2 inhibitors (SGLT2i) have shown promising benefits in the management of cardiovascular and renal outcomes in patients with type 2 diabetes mellitus (T2DM) and CKD [2]. The 2022 American Diabetes Association (ADA) and Kidney Disease Improving Global Outcomes (KDIGO) guidelines recommend SGLT2i as the first-line treatment for patients with an estimated glomerular filtration rate (eGFR) of ≥ 20 mL/min/1.73 m² and metformin for those with an eGFR of ≥ 30 mL/min/1.73 m² [3]. These drug groups have become the standard of care for CKD and cardiovascular health due to their renal and cardiovascular protective effects, independent of their glucose-lowering properties [4,5]. However, most studies have excluded patients receiving kidney replacement therapy (KRT) or kidney transplant (KT) recipients, leaving a gap in the literature regarding the safety, efficacy, and clinical outcomes of SGLT2i use in these populations [6-8]. Despite advances in dialysis treatment, patients on KRT continue to face a high risk of cardiovascular complications, compounded by the progressive decline in renal function. The management of CVD in this population is challenging, and there is an urgent need for interventions that can improve both cardiovascular health and residual kidney function (RKF). There is emerging evidence from post-hoc analyses and experimental and preclinical trials that supports the premise that SGLT2i may be equally effective in preventing cardiovascular and mortality outcomes in patients on KRT, whether on HD, peritoneal dialysis, or KT [9-12]. In addition, some observational studies suggest that SGLT2i may offer cardioprotective and nephroprotective effects in patients on KRT, including those on HD [13-16]. As of 2023, there are four major trials searching for an SGLT2i benefit in this population (NCT05687058, NCT05179668, NCT05141552, and NCT05374291) [17-20]. However, evidence remains preliminary, particularly regarding echocardiographic measures of cardiovascular function. This study is a pilot feasibility study given the novel echocardiographic focus in an HD population where such data are scarce.

Study objectives and endpoints

This study was primarily designed as an echocardiography-focused investigation to evaluate the cardiovascular effects of SGLT2 inhibition in HD patients. The renoprotective effects were assessed as exploratory outcomes.

Primary Objective

This study aims to determine whether empagliflozin 10 mg daily for 12 weeks reduces left ventricular filling pressure, assessed by the change in E/e′ ratio, in adults on maintenance HD.

Secondary Objectives

This study aims to assess the effect on cardiac structure and function (changes in left atrial volume index (LAVi), left ventricular end-diastolic pressure (LVEDP), left ventricular end-diastolic diameter (LVEDD)), to evaluate changes in systemic parameters (systolic blood pressure (SBP)/diastolic BP (DBP), New York Heart Association (NYHA) class, high-sensitivity C-reactive protein (hs-CRP)), and to explore the impact on residual kidney function, defined by 24-hour urine volume, and on albuminuria (albumin-creatinine ratio (ACR)) in patients with measurable urine output.

Safety Objective

This study aims to describe the incidence of adverse events (intradialytic hypotension, genitourinary infections, euglycaemic diabetic ketoacidosis, hypoglycaemia, and treatment discontinuations).

## Materials and methods

This prospective study was conducted between July and September 2024. All participants were then followed for a period of three months. The study enrolled CKD patients undergoing conventional HD three sessions per week, each lasting three to five hours, from the Haemodialysis Unit at Sayed Galal University Hospital, Al-Azhar University, Cairo, Egypt. Eligible participants were men and women aged 20-60 years with a body mass index (BMI) of ≤30 kg/m². The underlying aetiology of CKD was diabetes mellitus, with or without hypertension. All hypertensive patients were controlled on stable regimens that included angiotensin-converting enzyme inhibitors (ACEI) or angiotensin-receptor blockers (ARBs), calcium channel blockers, beta-blockers, or alpha-blockers. Five patients were treated with basal insulin, and none were receiving SGLT2i at enrolment. Dialysis vintage was limited to ≤12 months. In addition, patients were required to have clinical evidence of volume overload despite optimisation of dry weight, including moderate to severe exertional dyspnoea, orthopnoea, or paroxysmal nocturnal dyspnoea, as well as signs such as elevated jugular venous pressure and ankle oedema. Patients with CKD of non-diabetic or non-hypertensive origin; alternative causes of congestion, such as anaemia, hypoalbuminemia, or primary lung disease; or active genital or urinary tract infections were excluded. All patients received standardised HD per institutional protocol (four-hour sessions, Kt/V target ≥1.4) without significant prescription changes during the study. Volume status was managed per unit protocol. The mean interdialytic weight gain (IDWG) was 2.3 ± 0.7 kg, with a mean ultrafiltration volume of 2.1 ± 0.8 L per session, which remained consistent between baseline and follow-up assessments. Concomitant medications were maintained at stable doses. Written informed consent was obtained from all participants, and the protocol was approved by the institutional ethics committee. Comprehensive evaluations were performed at baseline and repeated after three months of therapy. Clinical assessment included the NYHA functional class, residual urine output, and vital signs such as blood pressure, pulse, and oxygen saturation at resting room air. We defined residual kidney function (RKF) a priori as urine output >100 mL/24 hours. For patients meeting this criterion, spot urine ACR was measured using samples with a minimum of 100 mL volume. Estimated glomerular filtration rate (eGFR) is reported as an exploratory biomarker only, recognising that standard eGFR equations are not validated in haemodialysis populations and do not represent measured glomerular filtration rate. Laboratory testing comprised serum creatinine, eGFR, hs-CRP, complete blood count, and uric acid levels. The erythropoietin and iron supplementation doses were stable. Urine ACR was measured from spot urine samples obtained during interdialytic periods. Only samples with a minimum volume of 100 mL were analysed to ensure reliability. Anuric patients (no measurable urine output) were excluded from ACR analysis but included in other study assessments. Transthoracic echocardiography was performed using a GE Vivid 9 machine with an M5S transducer (1.7-3.4 MHz). To minimise fluid-related artefacts, imaging was conducted 24 ± 2 hours after the preceding HD session. Ultrafiltration volumes were recorded for each session, with mean values of 2.2 ± 0.7 L at baseline and 2.1 ± 0.9 L at follow-up (p = 0.451), confirming stable volume removal between assessments. Patients with poor acoustic windows or significant arrhythmias during image acquisition were excluded. Echocardiographic measurements were obtained in accordance with the American Society of Echocardiography’s 2018 guidelines [21]. Two-dimensional parameters included LVEDD and left ventricular end-systolic diameter measured at the mitral leaflet tips, left ventricular mass index (LVMI) calculated using the linear method and normalised to body surface area, and left atrial diameter (LAD) measured at end-systole from the aortic root to the posterior atrial wall. Functional assessment was performed by calculating the LVEF using Simpson’s biplane method from apical four- and two-chamber views. LVEDP was estimated using the E/e′ ratio derived from septal and lateral tissue Doppler imaging, with values >14 considered elevated. The LAVi was determined using the area-length method at end-systole. To ensure accuracy, all measurements were averaged over three cardiac cycles and analysed offline by two independent blinded cardiologists using EchoPAC PC v203 (GE Healthcare Technologies, Inc., Chicago, Illinois, USA), achieving an intraclass correlation coefficient of 0.91. The intervention consisted of empagliflozin 10 mg administered once daily for three months. Dosing was initiated immediately after HD sessions to reduce the risk of intradialytic hypotension. All baseline assessments were repeated at the three-month follow-up using identical protocols. Statistical analyses were conducted using IBM SPSS Statistics for Windows, Version 26 (Released 2018; IBM Corp., Armonk, New York, United States) (or R version 4.2.2, depending on your software). Continuous variables were tested for normality using the Shapiro-Wilk test and expressed as mean ± standard deviation if normally distributed, or median with interquartile range if skewed. Categorical variables were presented as frequencies and percentages. Comparisons between baseline and post-treatment values were performed using a paired Student’s t-test for normally distributed variables and the Wilcoxon signed-rank test for non-normally distributed data. A two-tailed p-value of <0.05 was considered statistically significant, while trends were reported for 0.05 ≤ p ≤ 0.10. The E/e′ ratio was designated as the primary echocardiographic endpoint. Post-hoc calculation indicated >90% power (α = 0.05; effect size, d = 1.18) to detect the observed change of -2.4 ± 2.1 units.

## Results

Forty HD patients (24 males, 16 females; mean age of 49.1 ± 9.5 years) were enrolled between July and September 2024 and followed for three months. The cohort included 26 hypertensive patients and 11 diabetic patients (five insulin-dependent) as represented in Table 1.

**Table 1:** Baseline patient characteristics BMI: body mass index; NYHA: New York Heart Association; DM: diabetes mellitus (Values presented as mean ± SD, median (IQR), or n (%))

Parameter	Total cohort (n = 40)
Age (years)	49.08 ± 9.4
Male sex	24 (60%)
Weight (kg)	74.5 (68.0-82.0)
BMI (kg/m²)	25.85 ± 2.5
NYHA class	
-II	12 (30%)
-III	22 (55%)
-IV	6 (15%)
Dialysis vintage (months)	8.2 ± 2.1
Hypertension	26 (65%)
DM	11 (27.5%)

Clinical data

SGLT2i therapy was associated with significant clinical improvements in haemodynamic and functional parameters. There was a significant reduction in body weight and BMI after SGLT2i intervention (p = 0.013 and 0.022, respectively). There was also a significant reduction in SBP and DBP, as well as in heart rate (p < 0.001, 0.003, and 0.008, respectively). Oxygen saturation was significantly improved (p = 0.04). These changes coincided with improved NYHA functional class, where 30% of patients demonstrated at least one class improvement. Notably, 50% of NYHA class III patients (six patients) transitioned to class II, while trends towards improvement were observed even in class IV patients (Table 2).

**Table 2:** Changes in patient characteristics after SGLT2i intervention BMI: body mass index; NYHA: New York Heart Association; SBP: systolic blood pressure; DBP: diastolic blood pressure (Values presented as mean ± SD or median (IQR); paired t-test/Wilcoxon signed-rank test) *p < 0.05 (significant). p-values are exploratory. Mean/median differences presented with 95% confidence intervals. Effect sizes: Cohen’s *d* (parametric), rank-biserial *r* (nonparametric); OR: odds ratio for NYHA. NYHA improvement is defined as a reduction by ≥1 class

Parameter	Pre-intervention	Post-intervention	Mean difference (95% CI)	p-value	Effect size
Weight (kg)	74.5 (68.0-82.0)	72.8 (66.5-80.2)	-1.7 (-3.0 to -0.4)	0.013*	r = 0.28
BMI (kg/m²)	28.3 ± 3.1	27.8 ± 3.0	-0.5 (-0.9 to -0.1)	0.022*	d = 0.16
SBP (mmHg)	132 ± 14	125 ± 12	-7.0 (-10.2 to -3.8)	<0.001*	d = 0.55
DBP (mmHg)	78 ± 9	74 ± 8	-4.0 (-6.5 to -1.5)	0.003*	d = 0.47
O₂ saturation (%)	96 (94-97)	97 (95-98)	+1.0 (0.1 to 1.9)	0.041*	r = 0.21
Pulse (bpm)	72 ± 11	68 ± 10	-4.0 (-6.8 to -1.2)	0.008*	d = 0.38

NYHA class	Pre n (%)	Post n (%)	Absolute change	p-value	Effect size
Class II	22 (55%)	28 (70%)	+15%	0.008*	OR: 2.10 (1.21-3.65)
Class III	12 (30%)	6 (15%)	-15%	0.008*	OR: 0.48 (0.27-0.83)
Class IV	6 (15%)	4 (10%)	-5%	0.250*	OR: 0.67 (0.34-1.32)

Biomarkers and renal function

SGLT2i therapy demonstrated significant anti-inflammatory effects, with a 38% reduction in hs-CRP (p = 0.005). Serum uric acid decreased significantly (p = 0.012), while haemoglobin showed a trend towards improvement (p = 0.070). At baseline, 28 of 40 patients (70%) had preserved RKF (urine output >100 mL/24h). This declined to 25 patients (63%) at three-month follow-up. Among patients with measurable RKF, ACR decreased significantly from a median of 310 (200-440) to 230 (140-370) mg/g (p = 0.022). As an exploratory biomarker, eGFR showed a non-significant trend towards improvement (+0.8 mL/min/1.73m², p = 0.087 ).The modest eGFR increase (+0.8 mL/min/1.73m², p = 0.087) suggests potential renal protection even in advanced CKD (Table 3). Residual urine output was preserved in the majority of patients, with 28 of 40 (70%) having urine output >100 mL/24h at baseline and 25 of 40 (63%) at follow-up.

**Table 3:** Biomarker and renal function changes eGFR: glomerular filtration rate; ACR: albumin/creatinine ratio *p < 0.05 (paired t-test for parametric/Wilcoxon for non-parametric data). p-values are exploratory. Data presented as mean difference (95% CI) for key parameters. Effect sizes: Cohen's *d* (parametric), rank-biserial *r* (non-parametric). ‡ACR measured from spot urine samples with a minimum of 100 mL volume in patients with residual urine output >100 mL/24h 28 patients had measurable urine output at baseline, 25 at three-month follow-up

Parameter	Pre-intervention	Post-intervention	Mean difference (95% CI)	p-value	Effect size
eGFR (mL/min/1.73m²)	8.0 ± 2.0	8.8 ± 2.2	+0.8 (-0.1 to +1.7)	0.087*	d = 0.38
Haemoglobin (g/dL)	10.3 ± 1.1	10.8 ± 1.2	+0.5 (-0.1 to +1.1)	0.070*	d = 0.43
Serum uric acid (mg/dL)	7.4 ± 1.2	6.8 ± 1.1	-0.6 (-1.1 to -0.1)	0.012*	d = 0.52
ACR (mg/g)‡	310 (200-440)	230 (140-370)	-80 (-145 to -15)	0.022*	r = 0.35
hs-CRP (mg/L)	6.5(4.3-9.0)	4.0 (2.7-5.9)	-2.5 (-3.8 to -1.2)	0.005*	r = 0.57

Echocardiographic parameters

**Figure 1:**
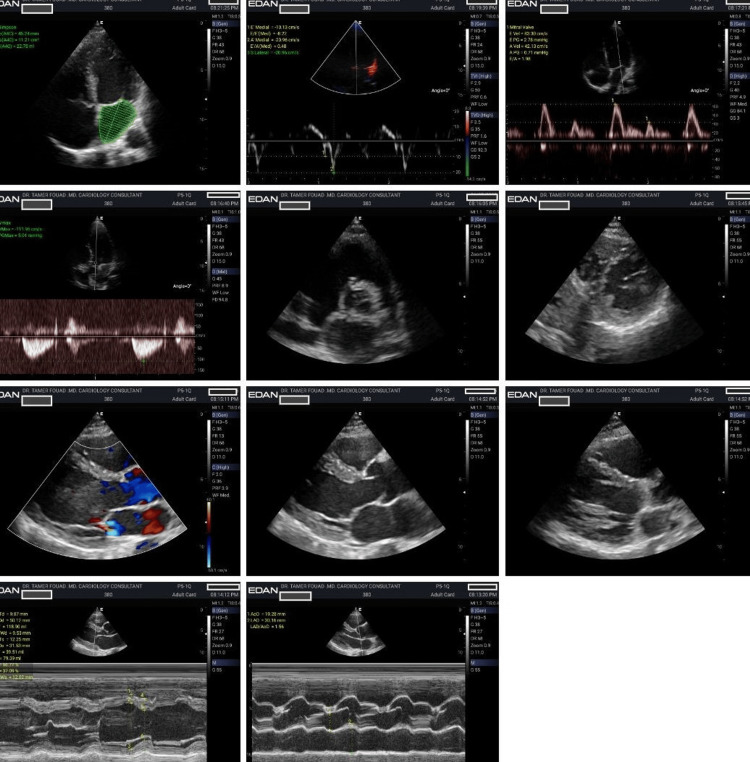
Baseline echocardiogram prior to SGLT2 inhibitor initiation SGLT2: sodium-glucose cotransporter 2 This figure represents a case with a baseline echocardiography demonstrating left atrial enlargement and diastolic dysfunction prior to treatment. The study is notable for a significantly dilated left atrium. The mitral inflow pattern and tissue Doppler indices show restrictive physiology, indicative of advanced diastolic dysfunction and high left atrial pressure

**Figure 2:**
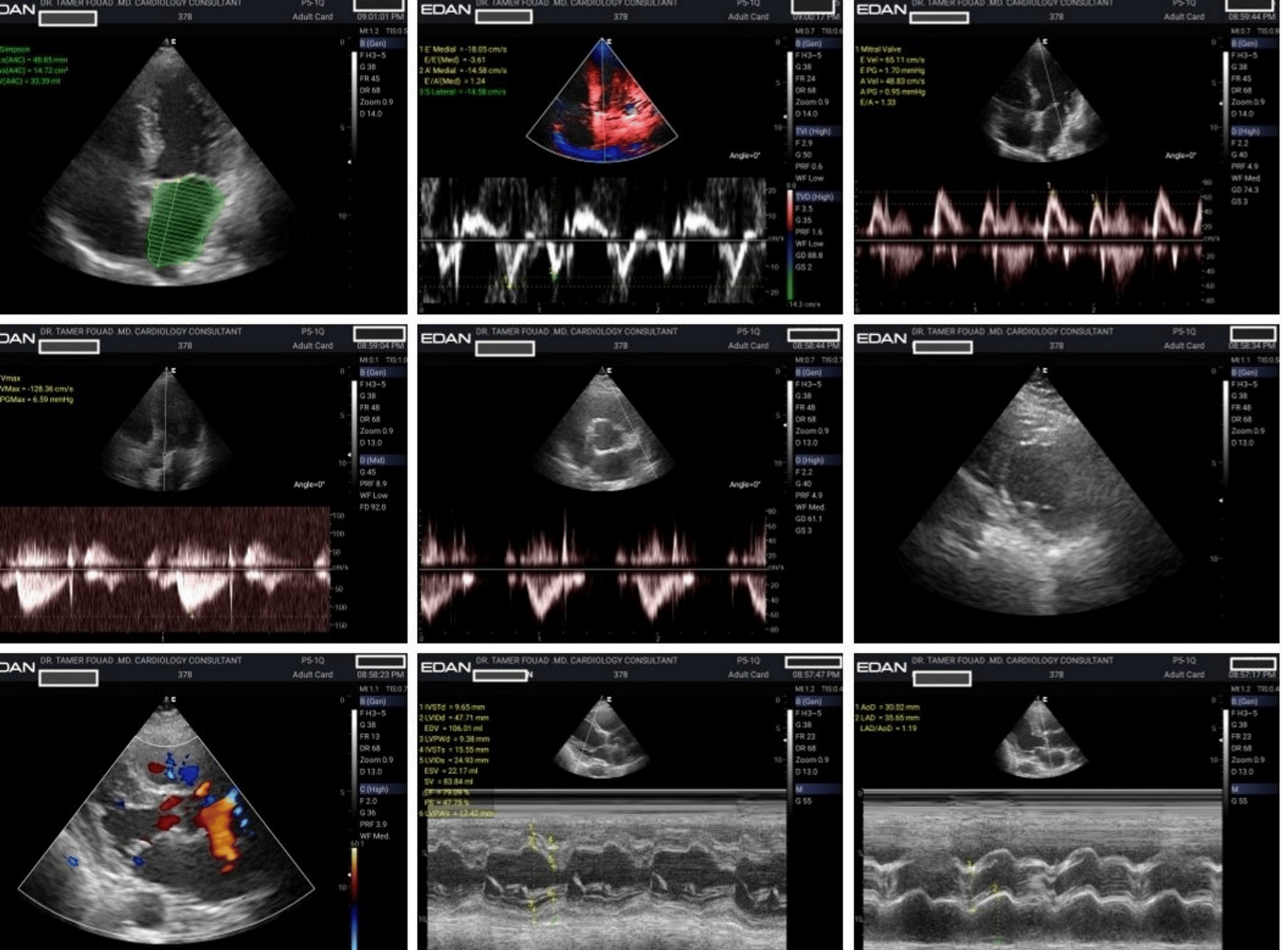
Improvement in echocardiographic parameters after SGLT2 inhibitor treatment SGLT2: sodium-glucose cotransporter 2 This figure shows an improvement in left atrial size and diastolic function after SGLT2 inhibitor therapy. A follow-up study shows a noticeable reduction in left atrial dimension and volume compared to baseline. The mitral inflow pattern and tissue Doppler indices also show a shift from a restrictive physiology towards a normal pattern, consistent with improved left ventricular compliance and lower filling pressures

SGLT2i therapy elicited profound improvements in diastolic function, with a 17% reduction in the E/e′ ratio (14.0 ± 2.2 to 11.6 ± 2.0, p < 0.001) and a 3.2 mmHg decrease in LVEDP, both indicating significantly lowered left ventricular filling pressures. Concurrent reverse remodelling was evidenced by significant improvements in both left atrial and ventricular metrics. The left atrium demonstrated substantial reverse remodelling, with a 7.6% reduction in its diameter (LAD: from 42.0 ± 3.5 mm to 38.8 ± 3.2 mm, p < 0.001) and an 11.8% decrease in its volume index (LAVi: from 38.0 ± 5.8 to 33.5 ± 5.3 mL/m², p < 0.001). Similarly, the left ventricle showed a 4.4% reduction in its end-diastolic dimension (LVEDD: from 51.8 ± 3.9 mm to 49.5 ± 3.6 mm, p < 0.001), while the ejection fraction remained stable (LVEF: from 55.5 ± 5.5% to 53.5 ± 5.2%, p = 0.110). These findings demonstrate consistent improvement across all echocardiographic markers of diastolic dysfunction, while maintaining preserved systolic function, a hallmark of effective haemodynamic unloading in volume-overloaded states (Table 4, Figures 1, 2). Inter-observer reliability was excellent (ICC for E/e′ = 0.91 (95% CI: 0.85-0.95)).

**Table 4:** Echocardiographic parameters LVEDP: left ventricular end-diastolic pressure; LAVi: left atrial volume index; LVEDD: left ventricular end-diastolic diameter; LVEF:  left ventricular ejection fraction *p < 0.05 (paired t-test for parametric/Wilcoxon for non-parametric data). p-values are exploratory except for the pre-specified primary endpoint (E/e′ ratio). Data presented as mean difference (95% CI) for key parameters. Effect sizes: Cohen's *d* (parametric), rank-biserial *r* (non-parametric)

Parameter	Pre-intervention	Post-intervention	Mean difference (95% CI)	p-value	Effect size
E/e' ratio	14.0 ± 2.2	11.6 ± 2.0	-2.4 (-3.1 to -1.7)	<0.001	d = 1.18
LVEDP (mmHg)	18.2 ± 3.0	15.0 ± 2.7	-3.2 (-4.2 to -2.2)	<0.001*	d = 1.13
LAD (mm)	42.0 ± 3.5	38.8 ± 3.2	-3.2 (-4.1 to -2.3)	<0.001*	d = 0.94
LAVi (mL/m²)	38.0 ± 5.8	33.5 ± 5.3	-4.5 (-6.3 to -2.7)	<0.001*	d = 0.82
LVEDD (mm)	51.8 ± 3.9	49.5 ± 3.6	-2.3 (-3.4 to -1.2)	<0.001*	d = 0.62
LVEF (%)	55.5 ± 5.5	53.5 ± 5.2	-2.0 (-4.5 to +0.5)	0.110*	d = 0.37

Safety outcomes

Empagliflozin was generally well-tolerated over the 12-week study period. Two patients (5%) experienced transient symptomatic hypotension managed with fluid administration without requiring HD session termination. One patient (2.5%) developed genital candidiasis that resolved with topical antifungal therapy. No cases of euglycaemic diabetic ketoacidosis, clinically significant hypoglycaemia, fractures, amputations, or volume depletion events occurred. Two patients required hospitalisation for unrelated reasons (one for vascular access infection, one for community-acquired pneumonia), with both events deemed unrelated to study medication by investigators. There were no study drug discontinuations due to adverse events.

## Discussion

This study provides novel echocardiographic evidence that SGLT2i ameliorate cardiovascular dysfunction in HD patients, a population with unique haemodynamic challenges. Our findings highlight the drug’s impact on left atrial (LA) mechanics, diastolic dysfunction, and volume status, key parameters assessable through transthoracic echocardiography and highly relevant to clinical management. These findings extend the known cardiorenal protective effects of SGLT2i to a population traditionally excluded from clinical trials, those on maintenance dialysis, with notable echocardiographic correlates of reduced congestion and reverse remodelling. The reduction in LVEDP (estimated by the E/e′ ratio) and LAVi reflects improved ventricular compliance and reduced pulmonary venous congestion. These changes correlate with the observed symptomatic relief (e.g. decreased orthopnoea, NYHA class) and align with SGLT2i’s known natriuretic and osmotic diuretic effects [22].

These findings are concurrent with those of El-Saied et al., who discovered the positive impact of SGLT2i on LA volume and functions, with an improvement in left ventricular diastolic and longitudinal functions [23]. Notably, the E/A ratio reversal (from restrictive to impaired relaxation pattern) in our cohort suggests enhanced early diastolic filling, a finding previously unreported in dialysis populations. While the LVEF non-significantly declined (remaining >50%) in our cohort, the reductions in LVMI and LVEDD indicate favourable remodelling [24], possibly due to an afterload reduction (from BP-lowering effects) [25] or anti-fibrotic mechanisms (supported by reduced hs-CRP) [26]. These findings suggest that SGLT2i facilitated reverse remodelling rather than impaired contractility. This parallels findings from HFpEF trials, such as EMPEROR-Preserved, where SGLT2i improved diastolic function without altering ejection fraction. Moreover, emerging data suggest these agents promote favorable cardiac remodeling even in conditions like transthyretin amyloid cardiomyopathy, where SGLT2i use alongside tafamidis was associated with improved cardiac function and structure in real-world analyses [27]. The reduction in LVEDD may indicate improved myocyte stretch and fibrosis modulation, consistent with preclinical data showing SGLT2i-mediated attenuation of inflammatory and fibrotic pathways [28].

The significant decrease in LAD is particularly noteworthy. LA dilation in HD patients is a strong predictor of mortality in end-stage renal disease [29], and its regression in our study suggests SGLT2i may modify this risk. In addition, LA size reduction could be translated into a lower atrial fibrillation risk in dialysis patients. These findings underscore the potential of SGLT2i to modify the trajectory of uremic cardiomyopathy, a critical yet understudied area in nephrology. Our study observed meaningful reductions in blood pressure alongside improved oxygen saturation, a constellation of findings pointing to SGLT2i-driven decongestion. These improvements point to SGLT2i-driven decongestion, potentially mediated by off-target effects on vascular tone and myocardial metabolism that improve ventricular loading conditions, rather than through direct diuretic action in this largely anuric population. These results echo those of Solomon et al. [30], who demonstrated similar haemodynamic benefits in heart failure patients, though our focus on a dialysis population adds novel insights into a traditionally understudied group. The echocardiographic data further unravelled the mechanisms behind these clinical improvements. The reduction in LVEDP, a critical marker of diastolic dysfunction, suggests improved cardiac filling dynamics, which may directly explain the alleviation of dyspnoea and orthopnoea in our cohort.

One of the most intriguing findings of our study was the observed numerical improvement in eGFR (though not statistically significant, p = 0.087), accompanied by preserved residual urine output and a meaningful drop in albuminuria. This triad challenges the long-held belief that SGLT2i lose efficacy in advanced CKD. While the glucose-lowering effects of these drugs diminish as kidney function declines, our data suggest a trend towards non-glycaemic organ protection, potentially mediated through alternative pathways such as tubuloglomerular feedback modulation or metabolic reprogramming. This aligns with recent work by Heerspink et al. [7] in the Dapagliflozin and Prevention of Adverse Outcomes in Chronic Kidney Disease (DAPA-CKD) trial, which showed kidney protection even at eGFR levels as low as 25 mL/min. Our results extend this observation to dialysis-dependent patients, a population excluded from most major trials, hinting that mechanisms like tubuloglomerular feedback modulation and metabolic reprogramming (e.g. ketone body utilisation) may remain active even in near-anuric states.

For HD patients, a population characterised by chronic inflammation and functional iron deficiency contributing to erythropoiesis-stimulating agent (ESA) hyporesponsiveness, the observed reductions in hs-CRP (p < 0.05) and trend towards haemoglobin improvement (p = 0.061) suggest potential dual modulation of inflammatory and erythropoietic pathways. While the haemoglobin change did not reach statistical significance, this trend aligns with post-hoc analyses from the EMPA-REG OUTCOME [31], where empagliflozin reduced inflammatory markers and transfusion requirements in diabetic kidney disease. The consistency of these findings, despite limited power, supports the hypothesis that SGLT2i may mitigate the chronic inflammation-erythropoietin resistance axis in advanced CKD [13]. The observed hs-CRP reduction and haemoglobin rise may reflect modulation of the interleukin-6-hepcidin pathway. Nonetheless, alternative explanations, such as improved infection control, dialysis adequacy, or changes in ESA therapy, may have contributed to these trends and should be acknowledged. For dialysis patients, who frequently experience both chronic inflammation and ESA-resistant anaemia, these biomarker improvements could potentially translate into clinically meaningful benefits, including fewer cardiovascular events and reduced dependence on costly ESAs.

The observed median albuminuria (ACR: 310 mg/g) was relatively moderate for a diabetic cohort, which may be explained by the advanced stage of kidney disease, where declining residual renal function and nephron mass lead to reduced total albumin excretion, a phenomenon consistent with the 'burn-out' of diabetic nephropathy. While our study was not powered to assess these hard endpoints, the mechanistic plausibility of these findings provides a strong rationale for larger, adequately powered trials specifically targeting these clinical outcomes in the dialysis population.

Limitations

The single-centre origin of our data offers internal consistency but warrants further multi-centre studies to enhance generalisability. Although the echocardiographic improvements we observed are promising and align with the known benefits of SGLT2i, confirming a causal relationship would require validation through a randomised controlled design. Furthermore, the three-month period was chosen to capture the early echocardiographic effects of treatment and was sufficient to demonstrate significant reverse remodelling; longer-term follow-up will be essential to confirm the durability of these structural improvements and to understand their relationship with clinical outcomes. Furthermore, our safety assessment was limited by the relatively short follow-up duration and small sample size, underscoring the need for larger trials with longer observation periods to fully characterise the safety profile of SGLT2i in haemodialysis patients.

## Conclusions

In this prospective pilot study of HD patients, empagliflozin was associated with significant improvements in echocardiographic parameters of diastolic function and reverse cardiac remodeling, alongside reductions in inflammation and albuminuria. These preliminary findings, observed over a three-month period and without a control group, suggest potential cardiorenal benefits of SGLT2 inhibition in end-stage kidney disease. Notably, to our knowledge, this represents one of the first prospective evaluations of empagliflozin in maintenance HD patients, a population largely excluded from major SGLT2i trials. The therapy demonstrated an acceptable short-term safety profile. Larger, randomised controlled trials with longer follow-up are warranted to confirm these exploratory results and establish efficacy and safety in this population.
